# Atorvastatin Attenuates Human Cardiac Fibroblast Activation, with Associated Changes in GATA4/MEF2C and Selected Fibrosis-Related microRNAs

**DOI:** 10.3390/ijms27094146

**Published:** 2026-05-06

**Authors:** Nikola Chomaničová, Adriana Adamičková, Zdenko Cervenak, Simona Valášková, Andrea Gažová, Jan Kyselovic

**Affiliations:** 1 Department of Pharmacology and Toxicology, Faculty of Pharmacy, Comenius University, 832 32 Bratislava, Slovakia; 2 5th Department of Internal Medicine, Faculty of Medicine, Comenius University, 813 72 Bratislava, Slovakia; 3 Institute of Pharmacology and Clinical Pharmacology, Faculty of Medicine, Comenius University, 813 72 Bratislava, Slovakia

**Keywords:** cardiac fibrosis, atorvastatin, myofibroblasts, GATA4, MEF2C, microRNAs

## Abstract

Cardiac fibroblast activation into α-smooth muscle actin (α-SMA)-expressing myofibroblasts is a central event in the progression of cardiac fibrosis. Therapeutic strategies capable of reversing or inhibiting this phenotypic transition are therefore of critical interest. Here, we explore associative changes in transcriptional and post-transcriptional regulators linked to fibroblast activation following atorvastatin exposure in primary human cardiac fibroblasts (HCFs). Atorvastatin treatment (10 µM) was associated with a reduction in α-SMA expression, consistent with decreased myofibroblast activation. This change co-occurred with reduced expression of the transcription factors GATA4 and MEF2C, which are implicated in cardiac cell identity and plasticity. Concurrently, atorvastatin treatment was associated with selective increase in specific fibrosis-related microRNAs, including miR-24, miR-26a, and miR-133a, whereas the expression of miR-21 and miR-23a remained unchanged. Together, these findings describe a coordinated pattern of transcriptional and post-transcriptional changes associated with atorvastatin exposure in HCFs, consistent with a shift away from the myofibroblast phenotype. These observations provide descriptive, hypothesis-generating insight into potential regulatory patterns associated with atorvastatin treatment, although further functional studies are required to establish causal relationships and translational relevance.

## 1. Introduction

Cardiac fibrosis, marked by excessive deposition of extracellular matrix (ECM), contributes to increased myocardial stiffness, disrupted electrical conduction, and progression toward heart failure [1]. A key cellular event in this process is the transition of human cardiac fibroblasts (HCFs) into contractile, ECM-secreting myofibroblasts, typically indicated by α-smooth muscle actin (α-SMA) expression [2]. This phenotypic shift is driven by coordinated transcriptional and post-transcriptional regulatory mechanisms. Among these, the transcription factors GATA-binding protein 4 (GATA4) and myocyte-specific enhancer factor 2C (MEF2C), although essential for cardiogenesis, are also involved in fibrotic gene expression programs in the adult heart [3,4,5,6]. Specifically, GATA4 and MEF2C act as crucial downstream effectors of the calcineurin/nuclear factor of activated T-cells (NFAT) signaling pathway, a major driver of the hypertrophic and fibrotic response in heart. Consequently, the targeted downregulation or inhibition of these transcription factors is considered a potentially beneficial strategy to limit fibrotic signaling [7,8]. In parallel, selected microRNAs (miRNAs) contribute to the post-transcriptional regulation of HCF transition, with miR-21 and miR-23a promoting profibrotic signalling [9,10,11] while miR-24, miR-26a, and miR-133a are associated with anti-fibrotic responses [12,13,14]. These miRNAs often intersect with broader regulatory frameworks, such as the IL-33/growth stimulation expressed gene 2 (ST2) signaling pathway or transforming growth factor-β (TGF-β)-dependent networks, which integrate cytokine-driven cues to modulate cardiac remodeling [15].

Statins exhibit well-documented pleiotropic effects. Beyond lowering cholesterol, they modulate intracellular signalling pathways, including inhibition of Ras homolog family member A (RhoA)/Rho-associated protein kinase (ROCK), which destabilises the actin cytoskeleton, and Phosphoinositide 3-kinase/protein kinase B/mechanistic target of rapamycin (PI3K/Akt/mTOR), which regulates cell survival and differentiation [16,17]. These actions contribute to their cardioprotective effects, including improved endothelial function, enhanced myocardial perfusion, and reduced adverse left ventricular remodelling [18,19,20,21,22]. Experimental and clinical studies further suggest that statins can limit fibrotic progression and promote reversal of the fibroblast-to-myofibroblast transition. Notably, such anti-fibrotic effects have been reported across multiple organ systems, supporting a broader role of statins in fibroblast biology [23,24,25]. In line with this, our previous work in patients with critical limb ischemia highlighted the clinical relevance of statins’ pleiotropic effects in conditions characterised by ischemia and fibrosis [26]. To better understand the molecular basis of these observations, in vitro models using primary human cells are essential, as they allow for the targeted investigation of regulatory networks under controlled, albeit simplified, conditions.

Despite these insights into the cardioprotective and anti-fibrotic actions of statins, it remains unclear which specific molecular regulators mediate these effects in cardiac fibroblasts. In particular, the potential role of atorvastatin in modulating the expression or activity of transcription factors GATA4 and MEF2C remains poorly understood in the context of cardiac fibrosis. Therefore, this study aimed to characterize the associations between atorvastatin treatment and changes in α-SMA expression, GATA4, MEF2C, and selected fibrosis-associated miRNAs in primary HCFs in vitro. By mapping these concurrent transcriptional and post-transcriptional changes, this study provides exploratory and descriptive insights into pleiotropic pathways through which atorvastatin may influence the fibrotic phenotype.

## 2. Results

### 2.1. Atorvastatin Does Not Induce Cytotoxicity in HCFs

**Figure 1:**
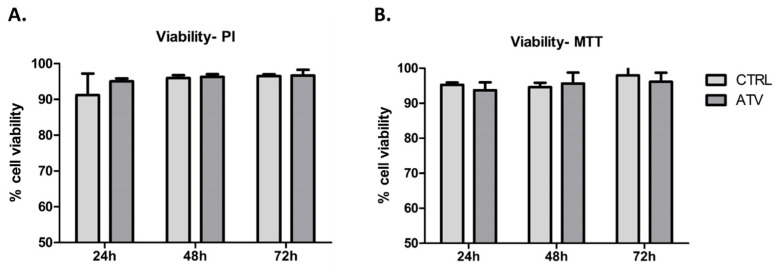
Viability of HCFs after treatment with atorvastatin, assessed by flow cytometry and MTT assay. (**A**). Flow cytometric analysis using PI staining showed that the proportion of viable (PI-negative) HCFs remained above 90% after 24, 48, and 72 h of atorvastatin (ATV) treatment (*n* = 3). (**B**). MTT assay confirmed sustained metabolic activity of HCFs under the same conditions, with cell viability consistently exceeding 90% at all time points (*n* = 8). Data are presented as mean ± SD. ATV = atorvastatin; CTRL = control.

Before assessing regulatory changes, we first evaluated whether 10 µM atorvastatin affects the viability of HCFs. Both flow cytometry (PI staining) and MTT assay demonstrated that cell viability remained above 90% throughout the 72-h treatment period (Figure 1), indicating that the selected concentration is well tolerated under the experimental conditions and does not compromise cellular integrity.

### 2.2. Atorvastatin Does Not Activate Apoptotic Signaling in HCFs

**Figure 2:**
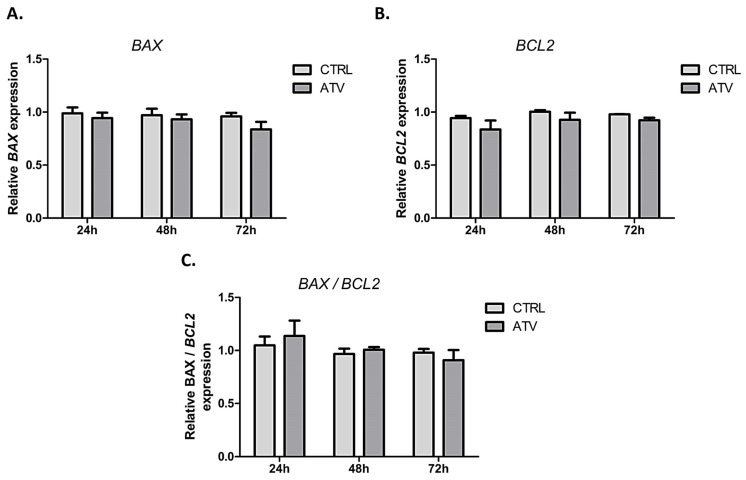
Relative mRNA expression levels of (**A**). *BAX*, (**B**). *BCL2*, and (**C**). *BAX/BCL2* ratio measured by qRT-PCR at 24, 48, and 72 h of atorvastatin treatment. Data are presented as mean ± SD (*n* = 3). ATV = atorvastatin, CTRL = control.

To further exclude activation of apoptotic pathways at the selected ATV concentration, we analyzed the expression of the pro-apoptotic marker BCL2-associated X protein (*BAX*) and the anti-apoptotic marker B-cell lymphoma 2 (*BCL2*). No significant changes were observed in either gene across all time points. In addition, the *BAX/BCL2* ratio remained stable, indicating that atorvastatin treatment does not induce apoptotic signaling under the applied conditions (Figure 2), further supporting the translational relevance of the used in vitro model.

### 2.3. Atorvastatin Preserves Mesenchymal Identity of HCFs

**Figure 3:**
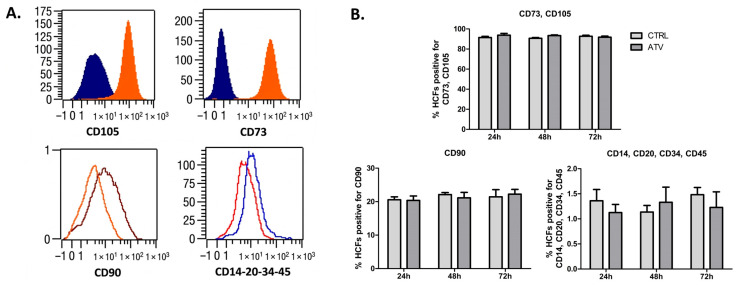
Mesenchymal identity of HCFs following atorvastatin treatment. (**A**). Representative flow cytometry histograms showing expression of surface markers after 48 h of atorvastatin exposure. Blue/dark histograms represent the isotype control, while orange/red histograms represent the atorvastatin-treated HCFs. HCFs remained positive for CD73 and CD105 and partially expressed CD90. Hematopoietic and endothelial markers (CD14, CD20, CD34, CD45) were consistently negative. (**B**). Quantitative summary of surface marker expression showing the average percentage of HCFs positive for mesenchymal markers (CD73, CD105, CD90) and negative for hematopoietic/endothelial markers. Data are presented as mean ± SD (*n* = 3). ATV = atorvastatin; CTRL = control.

We next assessed whether atorvastatin alters fibroblast identity. The mesenchymal surface marker profile of HCFs (CD73^+^/CD105^+^/CD90^+^/^−^) remained unchanged following treatment (Figure 3).

### 2.4. Atorvastatin Induces a Transient Change in Transcription of HMGCR

**Figure 4:**
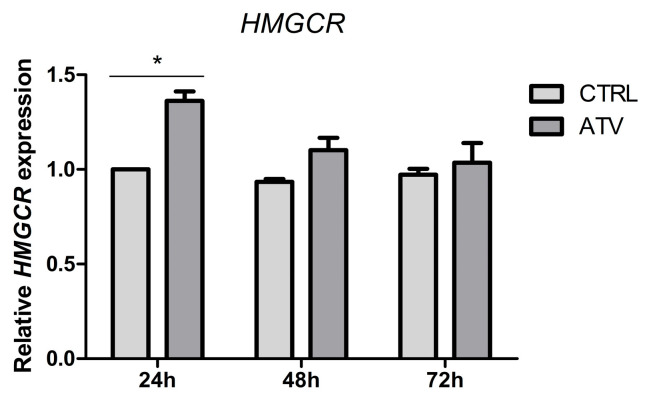
Relative mRNA expression levels of *HMGCR* measured by qRT-PCR at 24, 48, and 72 h of atorvastatin treatment, showing significant upregulation compared to control at 24 h (* *p* < 0.05). Data are presented as mean ± SD (*n* = 3). ATV = atorvastatin, CTRL = control.

We evaluated early metabolic regulation at the level of cholesterol biosynthesis as a positive control for statin activity. Expression of HMG-CoA reductase (*HMGCR*) was transiently increased at 24 h following atorvastatin treatment but returned to baseline levels at 48 and 72 h (Figure 4). This pattern is consistent with a transient, time-dependent compensatory feedback regulatory response of cholesterol metabolism to HMGCR inhibition [27,28].

### 2.5. Atorvastatin Attenuates Myofibroblast Activation Markers in Primary HCFs

**Figure 5:**
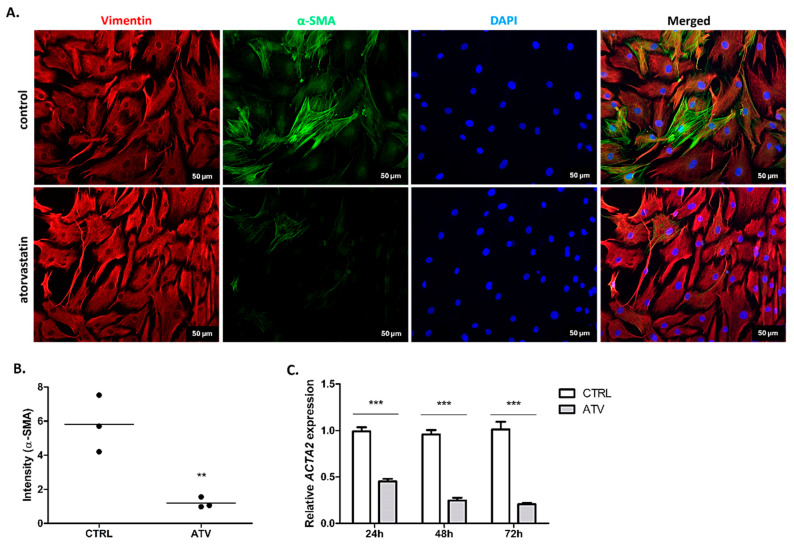
Immunocytochemical and qRT-PCR analysis of α-SMA expression in HCFs after atorvastatin treatment. (**A**). Representative immunofluorescence images showing α-SMA (green), vimentin (red), and nuclei counterstained with DAPI (blue). Images were captured at 20× magnification after 48 h of atorvastatin treatment. (**B**). Quantitative evaluation of α-SMA fluorescence intensity normalised to control demonstrating a significant decrease after 48 h of atorvastatin treatment (** *p* < 0.01). (**C**). Relative mRNA expression levels of *ACTA2* (α-SMA gene) measured by qRT-PCR at 24, 48, and 72 h of atorvastatin treatment, showing significant downregulation compared to control (*** *p* < 0.001). Data are presented as mean ± SD (*n* = 3). ATV = atorvastatin, CTRL = control.

The suppression of myofibroblast differentiation is a key indicator of anti-fibrotic efficacy, with α-SMA serving as a central marker of this phenotypic shift [29]. Atorvastatin treatment was associated with a significant reduction in α-SMA expression. Immunofluorescence analysis at 48 h revealed a marked decrease in the intensity and organization of the α-SMA signal (** *p* < 0.01) (Figure 5A,B). This phenotypic change was supported by a robust and sustained downregulation of *ACTA2* mRNA, which encodes α-SMA, that was observed at all measured time points (24, 48, and 72 h; *** *p* < 0.001) (Figure 5C). These findings suggest an association between atorvastatin treatment and reduced expression of myofibroblast activation markers in primary HCFs.

### 2.6. Atorvastatin Alters Expression of Key Transcription Factors in Associative Manner

**Figure 6:**
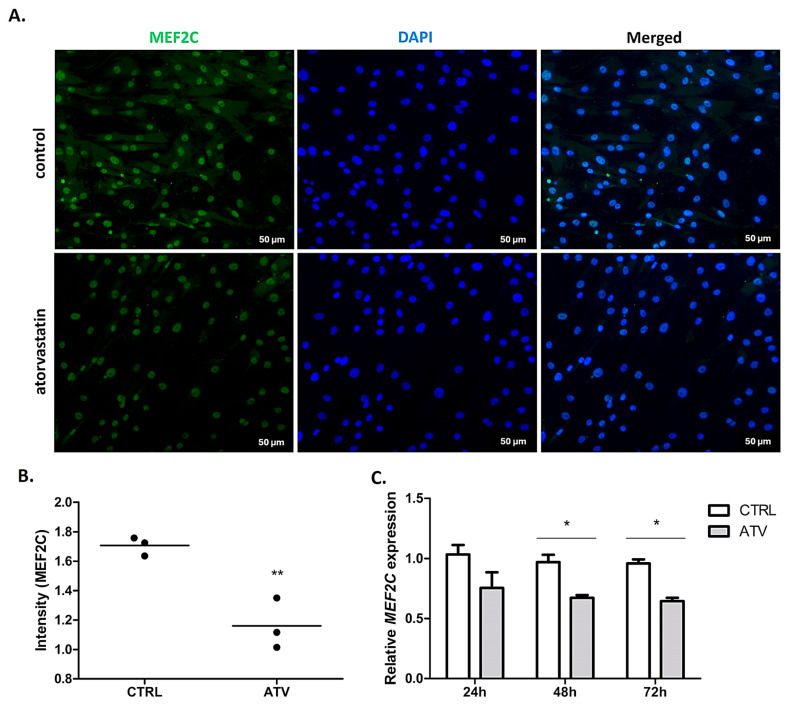
Immunocytochemical and qRT-PCR analysis of MEF2C expression in HCFs after atorvastatin treatment. (**A**). Representative immunofluorescence images showing MEF2C (green) localisation with nuclear counterstaining with DAPI (blue). Images were captured at 20× magnification after 48 h of atorvastatin treatment. (**B**). Quantitative analysis of MEF2C fluorescence intensity normalised to control levels revealing a significant reduction following 48 h of atorvastatin treatment (** *p* < 0.01). (**C**). Relative mRNA expression of *MEF2C* measured by qRT-PCR at 24, 48, and 72 h after atorvastatin treatment. Significant downregulation was observed at 48 and 72 h (* *p* < 0.05). Data are presented as mean ± SD (*n* = 3). ATV = atorvastatin, CTRL = control.

**Figure 7:**
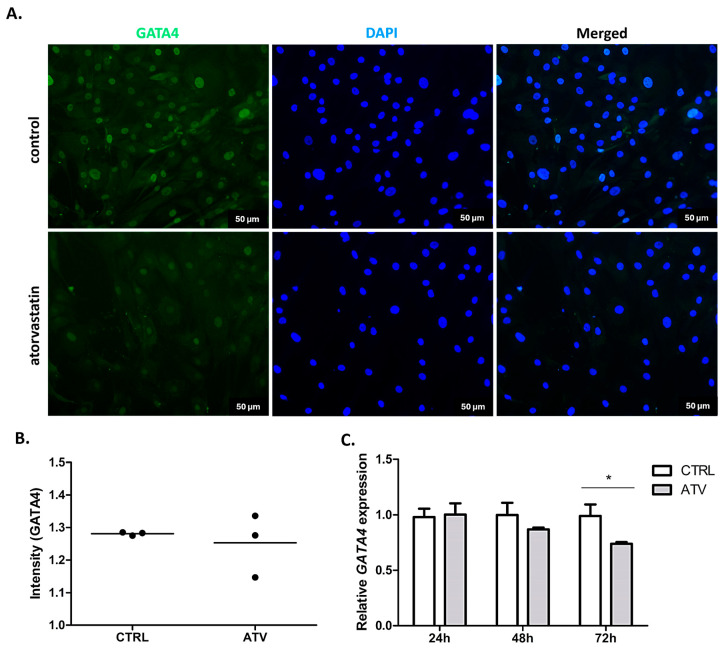
Immunocytochemical and qRT-PCR analysis of GATA4 expression in HCFs after atorvastatin treatment. (**A**). Representative immunofluorescence images showing GATA4 (green) localisation with nuclear counterstaining with DAPI (blue). Images were acquired at 20× magnification after 48 h of atorvastatin treatment. (**B**). Quantitative analysis of GATA4 fluorescence intensity normalised to control levels indicating no significant change after 48 h of atorvastatin treatment. (**C**). Relative mRNA expression of *GATA4* measured by qRT-PCR at 24, 48, and 72 h of treatment showing a significant decrease at 72 h (* *p* < 0.05). Data are presented as mean ± SD (*n* = 3). ATV = atorvastatin, CTRL = control.

To explore the potential upstream regulatory associations related to α-SMA expression, we examined the expression of transcription factors GATA4 and MEF2C, which are implicated in cardiac fibrotic signalling. Atorvastatin treatment was associated with a significant decrease in MEF2C expression, observed as a reduced immunofluorescence intensity (48 h; ** *p* < 0.01) and downregulated mRNA levels (48 and 72 h; * *p* < 0.05) (Figure 6). Similarly, *GATA4* mRNA levels were significantly reduced at 72 h (* *p* < 0.05) while protein-level analysis by immunofluorescence at 48 h did not show significant changes (Figure 7). This may suggest a potential temporal dissociation between transcript and protein-level responses, or that the reduction in *GATA4* mRNA represents a later transcriptional adjustment. The observed changes in GATA4 and MEF2C expression co-occurred with reduced α-SMA expression. However, these findings represent associative relationships and do not establish direct regulatory causality between transcription factor expression and myofibroblast activation markers.

### 2.7. Atorvastatin Modulates the Expression of Selected Fibrosis-Related MicroRNAs

**Figure 8:**
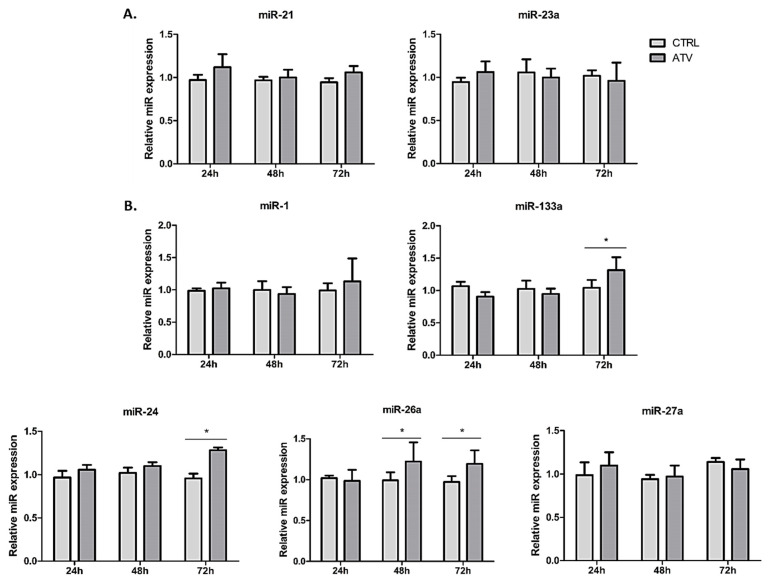
Expression of fibrosis-associated microRNAs in HCFs after treatment with atorvastatin. (**A**). Relative expression of miR-21 and miR-23a. (**B**). Relative expression of miR-1, miR-133a, miR-24, miR-26a, and miR-27a. Data are shown as mean ± SD (*n* = 3). Statistical significance was determined relative to the control (* *p* < 0.05). ATV = atorvastatin, CTRL = control.

In addition to the observed changes in transcription factors, we investigated whether atorvastatin treatment is associated with changes in miRNAs involved in post-transcriptional regulation of fibroblast activity. Atorvastatin treatment did not significantly alter the expression levels of profibrotic miR-21 and miR-23a (Figure 8A). However, it was associated with a selective increase in a subset of miRNAs linked to regulation of fibroblast activation (Figure 8B). Specifically, miR-26a expression increased at 48 and 72 h (* *p* < 0.05), while miR-24 and miR-133a were significantly upregulated at 72 h (* p < 0.05). Notably, the upregulation of these miRNAs occurred primarily at later time points, following the initial reduction in *ACTA2* mRNA. These data indicate an association between atorvastatin treatment and differential regulation of selected fibrosis-related microRNAs.

## 3. Discussion

This study explores a multi-level molecular signature associated with atorvastatin treatment in primary HCFs, with a particular focus on transcriptional and post-transcriptional correlates of fibroblast activation. Although canonical statin pleiotropic pathways such as RhoA/ROCK inhibition, PI3K/Akt signaling, and TGF-β/SMAD modulation were not directly evaluated in this study, previous studies have demonstrated that atorvastatin can attenuate profibrotic signaling through these pathways [19,30,31,32]. In our study, we observed that atorvastatin treatment is associated with a reduction in the myofibroblast marker α-SMA, consistent with prior reports that statins can attenuate myofibroblast differentiation and fibrotic remodelling in cardiac tissues [23,33,34,35]. However, it is important to consider that while α-SMA reduction reflects attenuated myofibroblast activation, it may also theoretically underline reduced cardiac repair capacity in certain pathological contexts [36]. Thus, while atorvastatin’s ability to limit α-SMA expression suggests a potential association with reduced fibroblast activation, it also raises important questions regarding the timing of its action.

GATA4 and MEF2C were selected for analysis based on their well-established roles in cardiac development, stress response, and transcriptional regulation of cardiac cell identity, as well as their reported involvement in fibroblast plasticity and remodeling processes [7]. Interestingly, the downregulation of GATA4 and MEF2C observed in our study contrasts with reports in cardiac progenitor or embryonic stem cells, where statins have been shown to induce these factors to promote cardiogenesis [37,38]. This discrepancy underscores the pronounced cell-type specificity of statin pleiotropy, whereby identical pharmacological stimuli may elicit divergent transcriptional responses depending on the cellular differentiation state. In differentiated HCFs, atorvastatin treatment is associated with the attenuation of transcriptional programs linked to fibrotic activation, whereas in progenitor populations, statins may facilitate lineage commitment [39].

This cell-specific role is further complicated by the fact that GATA4 and MEF2C are also central to cardiac cell identity and plasticity. Their forced overexpression, typically in combination with TBX5, is a well-established method to reprogram fibroblasts into induced cardiomyocytes [6,40,41]. Although TBX5 expression was not evaluated in our study, the observed reduction in GATA4 and MEF2C warrants a nuanced interpretation regarding the fibroblast phenotypic state. Recent evidence suggests that the functional consequences of GATA4 modulation are non-linear and highly dependent on the experimental system and baseline cellular environment. For instance, Yamada et al. (2025) demonstrated that overexpressing GATA4 in cardiac fibroblasts suppressed proinflammatory and fibrotic signatures in vivo [42]. While this might seem to contrast with our findings, it is essential to distinguish between the effects of artificial, high-level overexpression and the endogenous, pharmacological modulation observed in our model. The functional outcome of these transcription factors is highly sensitive to their absolute levels and the specific signaling context [43]. Therefore, rather than indicating a loss of cardiogenic plasticity [44], the concurrent reduction in α-SMA and these transcription factors may reflect an associative reconfiguration of the transcriptional landscape toward a less activated fibroblast phenotype. Notably, the observed reduction in *GATA4* mRNA levels did not consistently translate to changes at the protein level in our immunofluorescence assays. This discrepancy likely reflects divergent turnover dynamics between mRNA and protein or the inherent semi-quantitative limitations of fluorescence intensity measurements, and these findings should be interpreted as an associative transcriptional signature rather than definitive changes in protein abundance.

Complementing these transcriptional associations, the second aspect of this study involves post-transcriptional regulation. Atorvastatin selectively upregulated miR-24, miR-26a, and miR-133a, which are known to repress fibrotic pathways [13,45]. Notably, miR-24 and miR-133a increased most prominently at 72 h, whereas α-SMA was already reduced at 24 h. This temporal dissociation suggests a potential role for these miRNAs in maintaining rather than initiating the suppressed phenotype; however, this remains speculative, as no functional or temporal perturbation experiments were performed to directly test this sequence of events.

The selected miRNA panel was based on prior evidence linking these molecules to cardiac fibroblast activation and fibrosis-related pathways. In contrast to other well-characterized fibrosis-associated clusters, such as miR-143/145, which are primarily involved in smooth muscle differentiation, myofibroblast contractile identity, and cytoskeletal remodeling [46,47], miR-24, miR-26a, and miR-133a have been specifically implicated in modulating profibrotic signaling pathways. However, it is important to situate these changes within broader regulatory frameworks. For example, upstream cytokine pathways such as IL-33/ST2 signaling are known to intersect with TGF-β and MAPK-dependent remodeling networks [15], and may influence the miRNA responses observed here. In silico target prediction analysis using TargetScanHuman 8.0 [48] suggests that these upregulated miRNAs are predicted to target key profibrotic signaling and ECM remodeling: miR-26a targets collagen type I α1 (COL1A1), lysyl oxidase-like 2 (LOXL2), connective tissue growth factor (CTGF), and SMAD family member 1 (SMAD1); miR-24 targets lysyl oxidase (LOX) and platelet-derived growth factor receptor alpha (PDGFRA); and miR-133a targets transforming growth factor beta receptor 1 (TGFBR1). While these predictions suggest potential regulatory interactions, they remain computational and require future experimental validation.

Taken together, the convergence of these transcriptional and post-transcriptional changes suggests a coordinated response to atorvastatin treatment, shifting the cellular balance away from the myofibroblast phenotype. While the present study does not establish direct causality through loss-of-function experiments, these associations provide a more integrated view of the pleiotropic potential of statins in primary HCFs.

Limitations and future directions: This study has several limitations. First, a single concentration (10 μM) of atorvastatin was used. Although this exceeds typical clinical plasma levels, the findings should be interpreted strictly within the context of this in vitro experimental system. Second, the lack of ECM markers (e.g., COL1A1/3A1) remains a constraint; future studies should incorporate these to better support conclusions regarding anti-fibrotic activity. Third, while changes in GATA4 and MEF2C expression were clearly detected at the transcriptional level, quantitative protein-level validation using techniques such as Western blotting was not performed, limiting the strength of conclusions regarding protein abundance. Finally, this study focused on spontaneous, stiffness-driven myofibroblast activation during in vitro culture. Whether atorvastatin exerts similar effects under cytokine-induced profibrotic stimulation (e.g., TGF-β) remains an important question for future investigation.

## 4. Methods and Materials

### 4.1. Cell Culture and Treatment

Primary human cardiac fibroblasts (HCFs; Cat# 306-05F, Sigma-Aldrich, St. Louis, MO, USA) were cultured in T75 flasks using Cardiac Fibroblast Growth Medium (CF-medium; Cat# 316-500, Sigma-Aldrich) and maintained at 37 °C in a humidified 5% CO_2_ incubator. Primary HCFs are known to undergo spontaneous stiffness-driven activation when cultured on rigid plastic surfaces, resulting in basal α-SMA expression even in the absence of exogenous profibrotic stimuli [49]. The culture medium was replaced every 48 h. Upon reaching 70% confluence, HCFs at passage four were harvested using trypsin-EDTA (Cat. #59428C, Sigma-Aldrich), counted, and seeded at a density of 30,000 cells per 6 cm^2^ Petri dish for further experiments. Atorvastatin hemicalcium salt (Cat# Y0002315, Sigma-Aldrich) was dissolved in dimethyl sulfoxide (DMSO) and applied to the CF-medium at a final concentration of 10 µM (ATV). This concentration was selected based on our previous dose–response validation and is consistent with established in vitro models investigating pleiotropic effects of statins [19,50,51,52]. HCFs were treated for 24, 48, or 72 h. The control group (CTRL) consisted of cells cultured in standard CF-medium supplemented with 0.1% DMSO (vehicle control).

### 4.2. Flow Cytometry

The expression of surface antigens (CD105^+^, CD90^+^, CD73^+^, CD14^−^, CD20^−^, CD34^−^, CD45^−^) in the HCFs was analysed by flow cytometry using a MACSQuant Analyzer 10 flow cytometer (Cat# 130-096-343, Miltenyi Biotec, Bergisch Gladbach, Germany), MACSQuantify software version 2.13.3 (Miltenyi Biotec) and MSC Phenotyping Kit (Cat# 130-095-198, Miltenyi Biotec), following the manufacturer’s instructions. Cell viability was assessed using propidium iodide (Cat. #130-093-233, Miltenyi Biotec).

### 4.3. MTT Assay

HCFs (5000 per well) were seeded into a transparent 96-well plate in CF-medium; after 24 h, they were treated with ATV as described above. Cell viability was measured using the MTT (3-(4,5-dimethylthiazolyl-2)-2,5-diphenyltetrazolium bromide) assay (Cat# 11465007001, Roche, Basel, Switzerland) and a Varioskan LUX multimode microplate reader (Cat# VL0L00D0, Thermo Fisher Scientific, Waltham, MA, USA) according to the manufacturer’s instructions.

### 4.4. Immunofluorescence Staining

To investigate morphological changes, HCFs were seeded at a density of 10,000 cells per well onto glass coverslips pretreated with 0.1% gelatin (Cat. # G1393, Sigma-Aldrich) in a 10-well chamber slide for microscopy in CF medium. After 24 h to allow for cell attachment, cells were treated with ATV as described above for 48 h. Following treatment, cells were fixed in 4% paraformaldehyde and permeabilised with 0.1% Triton X-100 (Cat. # X100, Sigma-Aldrich) in phosphate-buffered saline (PBS) for 30 min. After blocking with 5% goat serum (Cat# G9023, Sigma-Aldrich) for 1 h, cells were incubated with primary antibodies against α-SMA (1:100 dilution, Cat# sc-130616, Santa Cruz Biotechnology, Dallas, Texas, USA), MEF2C (1:200 dilution, Cat# D80C1 XP^®^, Cell Signaling, Danvers, MA, USA), GATA4 (1:200 dilution, Cat# D3A3M, Cell Signaling) and vimentin (1:100 dilution, Cat# D21H3 XP^®^, Cell Signaling), followed by Alexa Fluor^®^ 568-conjugated goat anti-mouse IgG (1:200 dilution, Cat# A-11004, Invitrogen, Carlsbad, CA, USA) or Alexa Fluor^®^ 488 goat anti-rabbit IgG (1:200 dilution, Cat# A-11008, Invitrogen). Nuclei were counterstained with 300 nM DAPI (4′,6-diamidino-2-phenylindole, Cat# D1306, Invitrogen). Coverslips were mounted with ProLong™ Gold Antifade Mountant (Cat# P36930, Invitrogen) to preserve fluorescence. Fluorescent images were captured using a Ti-E microscope (Nikon Instruments, Tokyo, Japan) at 20× magnification. Fluorescence signal intensity was quantified using ImageJ software version 1.54p (National Institutes of Health).

### 4.5. qRT-PCR

qRT-PCR was used to analyse the gene expression of fibrosis-related transcription factors GATA4 and MEF2C, and microRNAs in HCFs. Total RNA was isolated using TRIzol™ Reagent (Cat# 15596026, Thermo Fisher Scientific) according to the manufacturer’s protocol. RNA and microRNA concentrations were measured using a Qubit™ 4 Fluorometer (Cat# Q33238 (https://www.thermofisher.com/order/catalog/product/Q33238#/Q33238 (accessed on 4 April 2026), Thermo Fisher Scientific) with the Qubit™ RNA HS Assay Kit (Cat# Q32852, Thermo Fisher Scientific) and Qubit™ microRNA Assay Kit (Cat# Q32880, Thermo Fisher Scientific), respectively. For reverse transcription, 1000 ng of total RNA and 60 ng of microRNA were used. Reverse transcription was carried out using the High-Capacity cDNA Reverse Transcription Kit with RNAse inhibitor (Cat# 4368814, Applied Biosystems, Foster City, CA, USA) or, for microRNAs, the miRCURY LNA RT Kit (Cat# 339340, Qiagen, Hilden, Germany), following the manufacturer’s instructions. The gene expression of *GATA4, MEF2C*, and *ACTA2* was assessed using TaqMan™ Universal Master Mix (Cat# 4304437, Applied Biosystems) with gene-specific TaqMan™ assays, using hGUSB as the reference gene. MicroRNA expression was analysed using the miRCURY LNA SYBR^®^ Green PCR Kit (Cat# 339345, Qiagen) with miR-16 and miR-454 as the reference microRNAs. All primers were validated to ensure the amplification of a single PCR product of the correct size, and no signal was detected when reverse transcription was omitted. qPCR reactions were conducted on a QuantStudio™ 5 Real-Time PCR System (Cat# A34322, Applied Biosystems), and relative gene expression was calculated using the ΔΔCt method [53]. Calculated normalised quantities were calibrated to the control group. The gene primer IDs and microRNA primer IDs utilised in this study are shown in Table 1.

### 4.6. Statistical Analysis

Statistical analysis was carried out using GraphPad Prism version 5.00 (GraphPad Software). Data are presented as the mean ± standard deviation (SD) from three independent experiments (biological replicates), each conducted in triplicate (technical replicates). The normality of the data distribution was assessed using the Shapiro–Wilk test. As the data did not meet the assumptions of normality, a nonparametric unpaired Mann–Whitney U test was used to compare the control and atorvastatin-treated groups. A *p*-value of < 0.05 (* *p*) was considered statistically significant.

## 5. Conclusions

In conclusion, atorvastatin treatment was associated with reduced markers of activation in primary HCFs coinciding with changes in the expression of α-SMA, the transcription factors GATA4 and MEF2C, as well as selected fibrosis-related microRNAs. These findings indicate that atorvastatin exposure is associated with concurrent changes in transcriptional and post-transcriptional regulators linked to cardiac fibroblast activation. This study provides descriptive, hypothesis-generating insight into regulatory patterns associated with statin exposure in HCFs; however, further functional studies are required to establish causal relationships.

## Figures and Tables

**Table 1:** Primer IDs used for the qRT-PCR analysis of selected genes and microRNAs.

Gene	Primer ID
hGUSB	Hs09939627
hMEF2C	Hs00231149
hGATA4	Hs00171403
hACTA2 (α-SMA)	Hs05005341
hBAX	Hs00180269
hBCL2	Hs00608023
hHGMCR	Hs00168352
microRNA	Primer ID
hsa-miR-21-5p	YP00204230
hsa-miR-23a-3p	YP00204772
hsa-miR-24-3p	YP00204260
hsa-miR-26a-5p	YP00206023
hsa-miR-27a-3p	YP00206038
hsa-miR-16-5p	YP00205702
hsa-miR-454-3p	YP00205663
hsa-miR-133a-3p	YP00204788
hsa-miR-1-3p	YP00204344

## Data Availability

The data that support the findings of this study are available upon request.

## Abbreviations

α-SMA—Alpha-Smooth Muscle Actin; Akt—Protein Kinase B; BAX—Bcl2-associated X protein, BCL2—B-cell lymphoma 2, ATV—Atorvastatin; COL1A1—collagen type I α1; CTGF—Connective Tissue Growth Factor; CTRL—Control; DAPI—4′,6-Diamidino-2-Phenylindole; DMSO—Dimethyl Sulfoxide; ECM—Extracellular Matrix; GATA4—GATA-Binding Protein 4; GUSB—Beta-Glucuronidase; HCFs—Human Cardiac Fibroblasts; HMG-CoA—3-hydroxy-3-methylglutaryl-coenzyme A; HMGCR—3-hydroxy-3-methylglutaryl-coenzyme A reductase, IL- interleukin, MAPK—mitogen-activated protein kinases, MEF2C—Myocyte Enhancer Factor 2C; miR—microRNA; MSC—Mesenchymal Stem Cells; mTOR—mammalian Target of Rapamycin; MTT—3-(4,5-Dimethylthiazol-2-yl)-2,5-diphenyltetrazolium bromide (assay); NFAT—nuclear factor of activated T-cells, NF-κB—Nuclear Factor Kappa-light-chain-enhancer of Activated B cells; PBS—Phosphate-Buffered Saline; PDGFRA—platelet-derived growth factor receptor alpha; PI—Propidium Iodide; PI3K—Phosphoinositide 3-kinase; RhoA—Ras homolog family member A; ROCK—Rho-associated protein kinase; SMAD1—SMAD family member 1, ST2—growth stimulation expressed gene 2, TGF-β—Transforming Growth Factor Beta; TGFBR1—transforming growth factor beta receptor 1.

## References

[1] Liu M., de Juan Abad B.L., Cheng K. (2021). Cardiac fibrosis: Myofibroblast-mediated pathological regulation and drug delivery strategies. Adv. Drug Deliv. Rev..

[2] Han M., Zhou B. (2022). Role of Cardiac Fibroblasts in Cardiac Injury and Repair. Curr. Cardiol. Rep..

[3] Cornwell J.D., McDermott J.C. (2022). MEF2 in cardiac hypertrophy in response to hypertension. Trends Cardiovasc. Med..

[4] Dittrich G.M., Froese N., Wang X., Kroeger H., Wang H., Szaroszyk M., Malek-Mohammadi M., Cordero J., Keles M., Korf-Klingebiel M. (2021). Fibroblast GATA-4 and GATA-6 promote myocardial adaptation to pressure overload by enhancing cardiac angiogenesis. Basic Res. Cardiol..

[5] Hong J.-H., Zhang H.-G. (2022). Transcription Factors Involved in the Development and Prognosis of Cardiac Remodeling. Front. Pharmacol..

[6] Huang P., Xu J., Keepers B., Xie Y., Near D., Xu Y., Hua J.R., Spurlock B., Ricketts S., Liu J. (2024). Direct cardiac reprogramming via combined CRISPRa-mediated endogenous Gata4 activation and exogenous Mef2c and Tbx5 expression. Mol. Ther.-Nucleic Acids.

[7] Heineke J. (2021). A NFAT decoy approach to inhibit cardiac hypertrophy. Pflügers Arch. Eur. J. Physiol..

[8] Zhu E., Zhang Z. (2025). Gata4 regulates cardiac fibrosis: A new mechanism to block fibroblast migration and expansion. Physiology.

[9] Ramanujam D., Schön A.P., Beck C., Vaccarello P., Felician G., Dueck A., Esfandyari D., Meister G., Meitinger T., Schulz C. (2021). MicroRNA-21–Dependent Macrophage-to-Fibroblast Signaling Determines the Cardiac Response to Pressure Overload. Circulation.

[10] Xia C., Cheng L., Zhao W., Chang A., Wang Z., Liu H., Pan X., Li W., Koji S., Li Z. (2025). LncRNA SYISL promotes fibroblast myofibroblast transition via miR-23a-mediated TRIOBP regulation. Cell. Mol. Life Sci..

[11] Zhou X., Xu H., Liu Z., Wu Q., Zhu R., Liu J. (2018). miR-21 promotes cardiac fibroblast-to-myofibroblast transformation and myocardial fibrosis by targeting Jagged1. J. Cell. Mol. Med..

[12] Mehjabin A., Kabir M., Micolucci L., Akhtar M.M., Mollah A.K.M.M., Islam S. (2023). MicroRNA in Fibrotic Disorders: A Potential Target for Future Therapeutics. Front. Biosci..

[13] Zhang W., Wang Q., Feng Y., Chen X., Yang L., Xu M., Wang X., Li W., Niu X., Gao D. (2020). MicroRNA-26a Protects the Heart Against Hypertension-Induced Myocardial Fibrosis. J. Am. Heart Assoc..

[14] Zhang Y., Wang Z., Lan D., Zhao J., Wang L., Shao X., Wang D., Wu K., Sun M., Huang X. (2022). MicroRNA-24-3p alleviates cardiac fibrosis by suppressing cardiac fibroblasts mitophagy via downregulating PHB2. Pharmacol. Res..

[15] Pentz R., Kaun C., Thaler B., Stojkovic S., Lenz M., Krychtiuk K.A., Zuckermann A., Huber K., Wojta J., Hohensinner P.J. (2018). Cardioprotective cytokine interleukin-33 is up-regulated by statins in human cardiac tissue. J. Cell. Mol. Med..

[16] Adamičková A., Gažová A., Adamička M., Chomaničová N., Valášková S., Červenák Z., Šalingová B., Kyselovič J. (2021). Molecular basis of the effect of atorvastatin pretreatment on stem cell therapy in chronic ischemic diseases—Critical limb ischemia. Physiol. Res..

[17] Samakova A., Gazova A., Sabova N., Valaskova S., Jurikova M., Kyselovic J. (2019). The PI3k/Akt pathway is associated with angiogenesis, oxidative stress and survival of mesenchymal stem cells in pathophysiologic condition in ischemia. Physiol. Res..

[18] Baehr A., Hinkel R., Kupatt C. (2020). Statins Make a Difference in Acute Myocardial Infarction: A Revival. J. Am. Coll. Cardiol..

[19] Du Y., Xiao H., Wan J., Wang X., Li T., Zheng S., Feng J., Ye Q., Li J., Li G. (2020). Atorvastatin attenuates TGF-β1-induced fibrogenesis by inhibiting Smad3 and MAPK signaling in human ventricular fibroblasts. Int. J. Mol. Med..

[20] German C.A., Liao J.K. (2023). Understanding the molecular mechanisms of statin pleiotropic effects. Arch. Toxicol..

[21] Lei X.-T., Pu D.-L., Shan G., Wu Q.-N. (2024). Atorvastatin ameliorated myocardial fibrosis by inhibiting oxidative stress and modulating macrophage polarization in diabetic cardiomyopathy. World J. Diabetes.

[22] Ovchinnikov A., Potekhina A., Arefieva T., Filatova A., Ageev F., Belyavskiy E. (2024). Use of Statins in Heart Failure with Preserved Ejection Fraction: Current Evidence and Perspectives. Int. J. Mol. Sci..

[23] Ross G., Kraft K., Emelyanova L., Rizvi F., Holmuhamedov E., Werner P., Tajik A.J., Jahangir A. (2016). Statin therapy reduces differentiation of ventricular fibroblast to myofibroblasts in human failing heart. FASEB J..

[24] Wang J., Wang Z., Xia F., Duan Q., Peng X. (2023). Atorvastatin reduces renal interstitial fibrosis caused by unilateral ureteral obstruction through inhibiting the transcriptional activity of YAP. Biochem. Biophys. Res. Commun..

[25] Yildirim M., Kayalar O., Atahan E., Oztay F. (2022). Atorvastatin attenuates pulmonary fibrosis in mice and human lung fibroblasts, by the regulation of myofibroblast differentiation and apoptosis. J. Biochem. Mol. Toxicol..

[26] Kyselovic J., Adamičková A., Gažová A., Valášková S., Chomaničová N., Červenák Z., Madaric J. (2024). Atorvastatin Treatment Significantly Increased the Concentration of Bone Marrow-Derived Mononuclear Cells and Transcutaneous Oxygen Pressure and Lowered the Pain Scale after Bone Marrow Cells Treatment in Patients with “No-Option” Critical Limb Ischaemia. Biomedicines.

[27] Göbel A., Breining D., Rauner M., Hofbauer L.C., Rachner T.D. (2019). Induction of 3-hydroxy-3-methylglutaryl-CoA reductase mediates statin resistance in breast cancer cells. Cell Death Dis..

[28] Lu H., Talbot S., Robertson K.A., Watterson S., Forster T., Roy D., Ghazal P. (2015). Rapid proteasomal elimination of 3-hydroxy-3-methylglutaryl-CoA reductase by interferon-γ in primary macrophages requires endogenous 25-hydroxycholesterol synthesis. Steroids.

[29] Ivey M.J., Tallquist M.D. (2016). Defining the Cardiac Fibroblast. Circ. J..

[30] Chen M., Li H., Wang G., Shen X., Zhao S., Su W. (2016). Atorvastatin prevents advanced glycation end products (AGEs)-induced cardiac fibrosis via activating peroxisome proliferator-activated receptor gamma (PPAR-γ). Metabolism.

[31] Gao G., Jiang S., Ge L., Zhang S., Zhai C., Chen W., Sui S. (2019). Atorvastatin Improves Doxorubicin-Induced Cardiac Dysfunction by Modulating Hsp70, Akt, and MAPK Signaling Pathways. J. Cardiovasc. Pharmacol..

[32] Kang Q., Kang M., Yang M., Fernando T. (2025). Inactivation of Notch1-TGF-β-Smads Signaling Pathway by Atorvastatin Improves Cardiac Function and Hemodynamic Performance in Acute Myocardial Infarction Rats. J. Vasc. Res..

[33] Kuo H.-F., Hsieh C.-C., Wang S.-C., Chang C.-Y., Hung C.-H., Kuo P.-L., Liu Y.-R., Li C.-Y., Liu P.-L. (2019). Simvastatin Attenuates Cardiac Fibrosis via Regulation of Cardiomyocyte-Derived Exosome Secretion. J. Clin. Med..

[34] Sun F., Duan W., Zhang Y., Zhang L., Qile M., Liu Z., Qiu F., Zhao D., Lu Y., Chu W. (2015). Simvastatin alleviates cardiac fibrosis induced by infarction via up-regulation of TGF-β receptor III expression. Br. J. Pharmacol..

[35] Yildirim M., Kayalar O., Atahan E., Oztay F. (2018). Anti-fibrotic effect of Atorvastatin on the lung fibroblasts and myofibroblasts. Eur. Respir. J..

[36] Campan M., Lionetti V., Aquaro G.D., Forini F., Matteucci M., Vannucci L., Chiuppesi F., Di Cristofano C., Faggioni M., Maioli M. (2011). Ferritin as a reporter gene for in vivo tracking of stem cells by 1.5-T cardiac MRI in a rat model of myocardial infarction. Am. J. Physiol. Circ. Physiol..

[37] Cianflone E., Torella M., Biamonte F., De Angelis A., Urbanek K., Costanzo F.S., Rota M., Ellison-Hughes G.M., Torella D. (2020). Targeting Cardiac Stem Cell Senescence to Treat Cardiac Aging and Disease. Cells.

[38] Yang C., Madonna R., Li Y., Zhang Q., Shen W.-F., McNamara K., Yang Y.-J., Geng Y.-J. (2014). Simvastatin-enhanced expression of promyogenic nuclear factors and cardiomyogenesis of murine embryonic stem cells. Vasc. Pharmacol..

[39] Xu X., Zhang L., Liang J. (2013). Rosuvastatin prevents pressure overload-induced myocardial hypertrophy via inactivation of the Akt, ERK1/2 and GATA4 signaling pathways in rats. Mol. Med. Rep..

[40] Ieda M., Fu J.-D., Delgado-Olguin P., Vedantham V., Hayashi Y., Bruneau B.G., Srivastava D. (2010). Direct reprogramming of fibroblasts into functional cardiomyocytes by defined factors. Cell.

[41] Zhao Y., Londono P., Cao Y., Sharpe E.J., Proenza C., O’rourke R., Jones K.L., Jeong M.Y., Walker L.A., Buttrick P.M. (2015). High-efficiency reprogramming of fibroblasts into cardiomyocytes requires suppression of pro-fibrotic signalling. Nat. Commun..

[42] Yamada Y., Sadahiro T., Nakano K., Honda S., Abe Y., Akiyama T., Fujita R., Nakamura M., Maeda T., Kuze Y. (2025). Cardiac Reprogramming and Gata4 Overexpression Reduce Fibrosis and Improve Diastolic Dysfunction in Heart Failure With Preserved Ejection Fraction. Circulation.

[43] Srivastava D., Ieda M. (2012). Critical factors for cardiac reprogramming. Circ. Res..

[44] Emelyanova L., Sra A., Schmuck E.G., Raval A.N., Downey F.X., Jahangir A., Rizvi F., Ross G.R. (2019). Impact of Statins on Cellular Respiration and De-Differentiation of Myofibroblasts in Human Failing Hearts. ESC Heart Fail..

[45] Wei P., Xie Y., Abel P.W., Huang Y., Ma Q., Li L., Hao J., Wolff D.W., Wei T., Tu Y. (2019). Transforming growth factor (TGF)-β1-induced miR-133a inhibits myofibroblast differentiation and pulmonary fibrosis. Cell Death Dis..

[46] Cordes K.R., Sheehy N.T., White M.P., Berry E.C., Morton S.U., Muth A.N., Lee T.-H., Miano J.M., Ivey K.N., Srivastava D. (2009). miR-145 and miR-143 regulate smooth muscle cell fate and plasticity. Nature.

[47] Jin J., Wang Z., Liu Y., Chen J., Jiang M., Lu L., Xu J., Gao F., Wang J., Zhang J. (2025). miR-143-3p boosts extracellular vesicles to improve the dermal fibrosis of localized scleroderma. J. Autoimmun..

[48] Agarwal V., Bell G.W., Nam J.-W., Bartel D.P. (2015). Predicting effective microRNA target sites in mammalian mRNAs. eLife.

[49] Felisbino M.B., Rubino M., Travers J.G., Schuetze K.B., Lemieux M.E., Anseth K.S., Aguado B.A., McKinsey T.A. (2023). Substrate stiffness modulates cardiac fibroblast activation, senescence and proinflammatory secretory phenotype. Am. J. Physiol. Circ. Physiol..

[50] Adamičková A., Chomaničová N., Gažová A., Maďarič J., Červenák Z., Valášková S., Adamička M., Kyselovic J. (2023). Effect of Atorvastatin on Angiogenesis-Related Genes VEGF-A, HGF and IGF-1 and the Modulation of PI3K/AKT/mTOR Transcripts in Bone-Marrow-Derived Mesenchymal Stem Cells. Curr. Issues Mol. Biol..

[51] Björkhem-Bergman L., Lindh J.D., Bergman P. (2011). What is a relevant statin concentration in cell experiments claiming pleiotropic effects?. Br. J. Clin. Pharmacol..

[52] Wang Q., Cui W., Zhang H.-L., Hu H.-J., Zhang Y.-N., Liu D.-M., Liu J. (2013). Atorvastatin Suppresses Aldosterone-induced Neonatal Rat Cardiac Fibroblast Proliferation by Inhibiting ERK1/2 in the Genomic Pathway. J. Cardiovasc. Pharmacol..

[53] Livak K.J., Schmittgen T.D. (2001). Analysis of Relative Gene Expression Data Using Real-Time Quantitative PCR and the 2^−ΔΔCT^ Method. Methods.

